# The SU.FOL.OM3 Study: a secondary prevention trial testing the impact of supplementation with folate and B-vitamins and/or Omega-3 PUFA on fatal and non fatal cardiovascular events, design, methods and participants characteristics

**DOI:** 10.1186/1745-6215-9-35

**Published:** 2008-06-10

**Authors:** Pilar Galan, Serge Briancon, Jacque Blacher, Sébastien Czernichow, Serge Hercberg

**Affiliations:** 1 UMR U557 Inserm, U1125 Inra, Cnam, Université Paris 13, CRNH IdF, F-93017 Bobigny, France; 2 EA 4003, Ecole de Santé Publique, Epidémiologie clinique, Faculté de Médecine, CHU Nancy, F-54000, Nancy, France; 3 Université Paris-Descartes, Faculté de Médecine, AP-HP, Hôtel-Dieu, Centre de Diagnostic et Thérapeutique, F-75004, Paris, France; 4 Département de Santé Publique, Hôpital Avicenne, 93017, F-Bobigny, France

## Abstract

**Background:**

During the last decades, many basic and clinical research have pointed to the role of B vitamins (folate, vitamins B6 and B12) and n-3 fatty acids as nutritional factors that might have a protective effect on the development of cardiovascular diseases (CVD).

**Methods/design:**

The SU.FOL.OM3 (SUpplementation with FOlate, vitamin B6 and B12 and/or OMega-3 fatty acids) trial is a randomized double-blind, placebo-controlled, secondary-prevention trial designed to test the efficacy of 5-methyl tetra-hydro-folates (5-MTHF) supplementation, in combination with vitamin B6 and B12 and/or n-3 fatty acids, at nutritional doses, on fatal and non fatal ischemic CVD in a 2 × 2 factorial design. A total of 2501 patients aged between 45 and 80 years who had a past history, in the previous year, of myocardial infarction (n = 1151) or instable angina pectoris (n = 711) or an ischemic stroke (n = 639) were included. Subjects have to be supplemented and followed up for five years. Daily supplementation comprised nutritional doses of 5-MTHF (560 μg), vitamin B6 (3 mg) and B12 (20 μg) and/or n-3 fatty acids (600 mg with an EPA:DHA ratio of 2:1). A factorial design 2 × 2 has been applied to investigate the separate effects of the B-vitamins, and the n-3 fatty acids, as well as their interaction as compared to the placebo.

The primary endpoint is a combination of myocardial infarction, ischemic stroke and cardiovascular death. Secondary endpoints are events of the composite endpoint taken separately, total mortality, and other cardiovascular events such as acute coronary syndromes, coronary revascularization, cardiac failure, arrhythmia...

**Conclusion:**

Baseline socio-demographic and medical characteristics of participants are totally comparable in the four randomized groups.

**Trial registration:**

Current Controlled Trials ISRCTN41926726

## Background

During the last decades, many basic and clinical research have pointed to the role of B vitamins (folate, vitamins B6 and B12) and n-3 fatty acids as nutritional factors that they might have a protective effect on the development of cardiovascular diseases.

The interest for B-vitamins is linked to the "homocysteine-hypothesis" of vascular disease [1,2]. Elevated plasma homocysteine levels have been related to a higher risk for coronary heart disease (CHD) and stroke, and homocysteine has been demonstrated to be lowered by folic acid and vitamin B12 supplementation [3], raising the prospect that adequate intake of B-vitamins could decrease the risk of CHD and stroke [4]. Several meta-analyses of prospective cohort studies evidenced that, after taking into account known risk factors, a lower homocysteine level was associated with a lower risk of CHD and a lower stroke risk [5-7].

Omega-3 fatty acids are thought to prevent cardiovascular diseases by their role in lowering levels of serum lipid [8], and by their anti-inflammatory, antithrombotic, anti-plateled-derived growth factor and antiarrhythmic properties. Several epidemiological studies from many countries suggested that an increased intake of dietary fish or fish oil rich in long-chain polyunsaturated n-3 fatty acids (PUFAs), eicosapentanoic acid (EPA) and docosahexaenoic acid (DHA), could decrease the incidence of cardiovascular diseases [9-13].

However, for B-vitamins as for n-3 fatty acids, epidemiological studies are mainly observational, which do not permit to conclude on a direct causal link between these nutrients and the risk of cardio and cerebrovascular diseases. Few years ago, some randomized trials have been launched to assess the effects of folate or n-3 fatty acids on cardiovascular outcome. Several CVD prevention large-scale B-vitamin supplementation trials were designed and initiated in the mid 1990's. The results of first of these large randomized trials has not provided clear evidence of any beneficial effect on CVD risk of lowering homocysteine concentration [14-17]. Possible reasons for the failure of these initial trials to demonstrate a beneficial effect might include: an inadequate number of participants, an insufficient duration of treatment, a reduction in the effect of B-vitamins by folic acid fortification; and finally, the true absence of effect on vascular risk. In retrospect, few of the completed homocysteine-lowering trials had sufficient statistical power to assess the predicted differences in risk suggested by the observational epidemiological studies. One of the possible reasons is that most of these trials were conducted in a country where folic acid-enriched flour is being compulsory. The consequence is a population-based homocysteine lowering effect and a potential power shortage of the above trials [15,17].

Regarding the effects of n-3 fatty acids on CVD outcomes, the totality of the data currently available suffered from limitations that make drawing firm conclusions difficult [18-20]. Indeed, the evidence to support cardiovascular benefits from n-3 PUFA intakes in patients with past history of cardiovascular diseases came from only one large randomized control trial (RCT) [21]. In some other trials, inconsistent antiarrhythmic effects and no significant overall effect on mortality have been observed in different types of patients with cardiovascular diseases [22-24]. Moreover, most of the available trials do not permit to conclude on the effects of specific fatty acids (25). Convergence of well-designed large RCTs that assess the effects of EPA and DHA on CVD outcomes with long follow-up periods, in different context are needed to conclude.

Finally, definitive evidence that supplementation with B-vitamins or n-3 fatty acids will lead to a reduced cardiovascular diseases morbidity and/or mortality is still scarce [25].

Taken all this information together, there was a need for a large double-blind placebo controlled randomized intervention trial evaluating the efficacy of supplementation with B-vitamins and n-3 fatty acids in nutritional doses with hard cardiovascular endpoints. Therefore, we decided to launch the SU.FOL.OM3 study (SUpplementation with FOlate, vitamin B6 and B12 and/or OMega-3 fatty acids) a secondary intervention trial designed to test the efficacy of daily supplementation with 5-methyl-THF/vitamin B12/B6 and/or n-3 polyunsatured fatty acids to prevent fatal and non fatal coronary heart disease and stroke. The present report describes design, implementation and baseline characteristics of participants of this 5-year cohort study, which started in 2003 in France.

### Study design

The SU.FOL.OM3 study is a double-blind randomized placebo-controlled secondary intervention trial in which participants are supplemented daily for a period of 5 years with nutritional doses of 5-methyl-THF (560 μg), vitamin B6 (3 mg) and B12 (20 μg) and/or n-3 fatty acids supplements (600 mg with an EPA:DHA ratio of 2:1). A 2 × 2 factorial design has been used to investigate the separate effects of the B-vitamins (S1), and the n-3 fatty acids (S2) as well as their interaction. B-vitamins placebo (P1) and omega-3 fatty acid placebo (P2) pills have been developed to respct the blindness of the trial. Participants have been randomly allocated to the following treatment groups: 5-methyl THF, vitamin B6 and B12 and a "omega-3 fatty acid placebo" (S1P2); n-3 fatty acids and a "5-methyl THF, vitamin B6 and B12 placebo" (S2P1); 5-methyl THF, vitamin B6 and B12 and omega-3 fatty acids (S1S2); the two placebos (P1P2).

The primary objective of this experimental epidemiological study is to test the hypothesis that supplementation with 5-methyl THF, vitamin B6 and B12 and/or omega-3 fatty acids prevents death from cardiovascular causes, recurrence of ischemic events in patients who already have experienced a coronary or cerebrovascular event. Secondary objectives are to prevent death for any cause and hospitalization for events of the composite endpoint taken separately and other cardiovascular events.

The protocole has been approved by the ethical committee for studies with human subjects named the "Comité Consultatif pour la Protection des Personnes se prêtant à la Recherche Biomédicale" (CCPPRB n° 1933) of Paris-Cochin, and the "Comité National Informatique et Liberté" (CNIL n° 901230) for keeping all medical information confidential and anonymous. All patients provided written informed consent.

### Population, sampling

The study cohort involves patients with a history of cardiovascular diseases recruited from all around France.

Estimation of sample size and length of follow up were based on models including the specification of the incidence of the trial outcomes in France (incidence of cardio and cerebro-vascular disease in secondary prevention), estimated adherence to taking the supplement capsules, rate of loss to follow-up and statistical parameters. Our goal is to be able to detect relatively weak effects with an alpha risk of 5 % and a power of 90%, testing a one-sided effect. Even if we will check an interaction between B-vitamins and n-3 fatty acids, a priori, we do not expect such an interaction and therefore we will not take it into account while estimating the sample size.

#### Risk reduction in each group

Our hypothesis is to observe a risk reduction of fatal and non fatal coronary and cerebrovascular events due to each intervention of 10%, equivalent to a risk ratio of 0.9 in group S1P2 and S2P1 and a risk of 0.81 in group S1S2 as compared to the risk of the P1P2 group.

#### Baseline risks in each group

The baseline risk of the P1P2 group was estimated to 0.087. The baseline risk of the S1P and S2P group is thus expected 0.078 (0.087 × 0.9).

#### Risk reduction in combined groups

To estimate the effect of S1 we will compare the combined group S1P2 and S1S2 with the combined group P1P2 and S2P1. The risk reduction in group S1P2 and S1S2 is thus estimated at 14.5% ([10% in group S1P2 and 19% in group S1S2]/2).

#### Baseline risk in combined groups

The baseline risk in group P1P2 and S2P1 is: 0.0826 (= [0.078 + 0.087]/2). To estimate the effect of S2 we will compare group S2P1 and S1S2 with group P1P2 and S1P2, the baseline risk in group P1P2 and S1P2 is also: 0.0826 (= [0.078 + 0.087]/2).

For the calculation of the necessary number of patients we have taken into account the comparison of exponential survival distributions, and a follow up of 5 years for each included subjects. With these parameters we calculated that a number of 1188 subjects are necessary in each combined group, for a total of 2376 patients. Taking into account possible dropout, we aim at including 2500 subjects in the trial.

### Recruitment and follow-up

First recruitment of subjects have been done through a network of 392 cardiologists and neurologists, all around France, working either in general hospitals, independently or in rehabilitation centers.

Follow-up time was defined by the time from the baseline visit until the first event date (for those who had an event); patients without event will be censored at the end of follow-up.

Clinicians had the responsibility to give potential study participants (i.e. patients that meet the eligibility criteria) the first information about the study protocol. They explained that participation included taking daily freely provided supplements. In addition, they explained that 25% of all participants received a complete placebo treatment. The clinicians also explained that despite the fact that they might receive only placebo, all participants benefit from a free annual biological check-up.

The clinicians offered a short questionnaire to all patients willing to participate. The questionnaire asked for 1) information by which inclusion of patients was checked, 2) the patients address and 3) permission to give the questionnaire's information to the SU.FOL.OM3 team. Once the clinicians have obtained a completed questionnaire from potential participants, the SU.FOL.OM3 team contacted the patient in order to verify his/her eligibility, to collect demographic, life-style and present and past health informations and to arrange an appointment.

Then patients were randomly assigned to a treatment group by means of a computerized system. The randomization was stratified for age in 3 classes (44–54, 55–64, 65–80), sex, and the type of pathologies at enrolment and region of residence. Once participants were randomized, they underwent a yearly visit and received their supplementation (enough for 1 year) in a package carrying their identification number.

### Inclusion criteria

The participants should be 45 – 80 years at baseline. Participants should have experienced a coronary or cerebral ischemic acute event 1 to 12 months before inclusion. A coronary or cerebral event was defined as: 1) myocardial infarction; 2) coronary syndrome without myocardial infarction; 3) an ischemic cerebral vascular accident.

- To fulfill the cerebrovascular disease criteria, the patients must have had a previous documented ischemic stroke (classical clinical criteria plus cerebral imaging: computed tomography or magnetic resonance imaging) with a Rankin [26] score < 3 at 3 months (ICD-10 codes: I63.0–I63.9). Transient ischemic attack does not represent an inclusion criteria.

- To fulfill the coronary acute event criteria [27], the patients must have had either a previous documented myocardial infarction (ICD-10 codes: I21.0–I21.9), defined by at least 2 of the following :

◦ Typical chest pain,

◦ Electrocardiographic modifications related to myocardial infarction,

◦ Cardiac enzymes increase

Or an acute coronary syndrome without myocardial infarction (ICD-10 codes: I20.0–I20.1), defined by the 2 of the 3 following criteria:

◦ Typical chest pain;

◦ Electrocardiographic modifications related to acute coronary syndrome or coronary artery disease documented in the management of the acute coronary syndrome with an angiographic coronary stenosis over 50%;

◦ Evidence-based previous coronary artery disease defined by previous angina pectoris (chest pain precipitated by exertion and relieved by rest or nitrates) confirmed by coronary angiography or a positive exercise test, previous myocardial infarction, typical sequelae on electrocardiography, previous coronary percutaneous transluminal angioplasty, and/or previous coronary bypass surgery.

Were not included:

- patients that are incapable of understanding the study protocol or who refuse to sign the informed consent;

- patients with a pathology that might interfere with homocysteine or omega-3 fatty acid metabolism, in particular those that use methotrexate for the treatment of a cancer or rheumatoid arthritis;

- chronic renal failure (plasma level of creatinine > 200 μmol/L or creatinine clearance < 40 ml/min);

- patients with a non-cardiovascular pathology with a suspected survival time less than the 5 years period of the study (solid cancer, evolved dementia, leukemia, etc.);

- patients taking treatment with B vitamins or omega-3 fatty acids.

### Outcome

Fatal and non fatal cardiovascular events, and all causes mortality were reported. Research assistants verified all information by checking medical records, hospital discharge forms, death certificates, and autopsy data. A coding form is used to classify all the cardiovascular events. Two Medical Committees were established, comprising epidemiologists, cardiologists, internists, pathologists and neurologists. Members of these committees were unaware of study-group assignments. Their task was to provide a validation of cardiovascular and stroke events during follow-up, in addition to mortality causes, according to the International Classification of Diseases, 10^th ^edition (ICD-10) [28].

Deceased subjects were confirmed from the mortality records of the Institut National de Statistiques et d'Etudes Economiques. A patient of our cohort was considered to be deceased when he had the same first name, last name, sex, and date of birth as a person recorded in the Institut National de Statistiques et d'Etudes Economiques mortality records during the period of follow-up. These data were provided by the Department of Mortality of the Institut National de la Santé et de la Recherche Médicale (INSERM).

### Endpoints

The primary endpoint is the composite of the first major cardiovascular event, which was defined as nonfatal myocardial infarction (definition above), stroke (definition above), or death from cardiovascular disease. Death from cardiovascular cause was defined as:

- fatal myocardial infarction;

- fatal stroke, ischemic, hemorrhagic or of unknown etiology;

- sudden death that was defined as a witnessed death that occurred within 1 h after the onset of acute symptoms, with no evidence that violence or accident played any role in the fatal outcome;

- fatal aortic dissection, fatal cardiac failure, and any fatal event defined as from cardiovascular cause by the medical committee.

### Secondary endpoints

Secondary endpoints are events of the composite endpoint taken separately, total mortality, and other cardiovascular events:

- each component of the composite primary endpoint taken isolately;

- acute coronary syndrome without myocardial infarction;

- resuscitate sudden death;

- coronary bypass surgery;

- percutaneous transluminal angioplasty;

- acute cardiac failure;

- ventricular arrhythmia;

- supra ventricular arrhythmia;

- any cardiac surgery;

- transient ischemic attack;

- deep vein thrombosis;

- pulmonary embolism;

- carotid surgery or carotid percutaneous transluminal angioplasty;

- peripheral arterial surgery or percutaneous transluminal angioplasty;

- any vascular procedure.

### Treatment

Study supplements were given as 2 capsules per day. The supplements were provided in a package containing 53 blisters of 2 × 7 capsules, each corresponding to one week of both treatments.

It was planned that participants will betaking the supplements for 5 consecutive years. At the first appointment participants were given supplements for the first treatment year. The supplements for the following 4 years will be supplied each year during the annual clinical and biological check-up of the participants.

The supplements consist of soft gelatin capsules. These capsules either contain the B-vitamins, the n-3, or the placebo substance. The placebo capsules look and taste identical to the active supplements. The raw material for the n-3 capsules have been provided without charge by Pierre Fabre, Merck Eprova AG provided the 5-methylTHF and Roche Laboratory the B6 and B12-vitamins. The gelatin capsules are manufactured by Catalent Pharma Solutions (Beinheim, France; Swindon, UK). Packaging of the capsules has been done under the responsibility of Klocke (Weingarten, Germany). These sponsors were not involved in the design, execution, analysis, or reporting in the trial results.

### Follow-up examination

Participants were given the standard therapy for their disease having led to the inclusion and each subject underwent a yearly visit consisting in a physical and biological monitoring check-up in 257 centers (hospitals or heath centers). Every six months after enrollement, a questionnaire is mailed to each participant. Subjects were asked about study outcomes (diseases, hospitalizations, etc.) and compliance. Those who do not return the questionnaire were interviewed by telephone by dedicated study physicians. Moreover, each year a simple follow-up questionnaire is mailed to to the participants allowing to collect data on incident health events and on the evolution of cardiovascular and lifestyle factors (smoking, dietary habits, physical activity, etc.).

The general practioners or cardiolologists or neurologists who follow the SU.FOL.OM3 subjects are requested to report any relevant information on health-events of the SU.FOL.OM3 patients.

In the blood that is drawn during the first appointment (baseline: T0) the following parameters were determined : total cholesterol, triglycerides, HDL-cholesterol, LDL-cholesterol, fasting glucose were measured by enzymatic method with the use of C8000 System Abbott (Rungis, France); the fatty acid composition of plasma lipids was determined by gas-chromatography, plasma homocysteine, serum folate and vitamin B12 as well as RBC folates were measured by a competitive immunoassay with direct chemiluminescence detection, pyridoxal 5'-phosphate (circulating form of vitamin B6) was determined in plasma by high-performance liquid chromatography; the polymerase chain reactions for 677 C>T and 1298 C mutations in the gene encoding for MTHFR were performed according to Frost and van der Put methos respectively.

Each year, for the subsequent years approximately on the anniversary date of baseline (T1, T2, T3, and T5), plasma homocysteine, folate, vitamin B12 and pyridoxal phosphate concentrations, erythrocyte concentration of folate, total cholesterol, triglycerides, HDL-cholesterol, LDL-cholesterol, and fasting glucose were planned to be measured for the whole sample and fatty acids profiles in a subsample. A biobank has been set up at baseline.

## Participant characteristics at baseline

2501 patients were included in the SU.FOL.OM3 cohort (1987 men and 514 women): age was 61.5 + 0.2 (m ± SD) years for men and 63.7 + 0.4 years for women; 1151 participants have experienced a myocardial infarction ; 711 an acute coronary syndrome without myocardial infarction and 639 an ischemic stroke.

X persons presented blood level for B-vitamins corresponding as values observed in subjects taking B-vitamins supplements or treatment. As this was an exclusion criteria, those randomized subjects were considered in condition of protocol violation and were excluded for analysis.

Principal characteristics, at the start of intervention, of participants contributing to analysis, are presented, for the 4 groups in Table 1.

**Table 1:** General characteristics of sample at baseline by group *

	Intervention groups			
	A	B	C	D

n	620	633	622	626

Age (years)	61.5 ± 9.3	61.4 ± 9.3	61.4 ± 8.7	61.4 ± 8.9
Gender				
- Men (%)	492 (79.3)	501 (79.1)	497 (79.9)	497 (79.4)
- Women (%)	128 (20.7)	132 (20.9)	125 (20.1)	129 (20.6)
Inclusion criteria				
- myocardial infarction (%)	279 (45.0)	301 (47.5)	288 (46.3)	283 (45.2)
- unstable angina (%)	176 (28.4)	184 (29.1)	168 (27.0)	183 (29.2)
- stroke (%)	165 (26.6)	148 (23.4)	166 (26.7)	160 (25.6)
Level of education (%)				
Elementary school	374 (61.9)	364 (59.0)	369 (60.6)	376 (61.0)
Secondary school	138 (22.9)	145 (23.5)	151 (24.8)	136 (22.1)
University or equivalent	92 (15.2)	108 (17.5)	89 (14.6)	104 (16.9)
Smoking (%)				
Current smokers	69 (11.4)	69 (11.3)	67 (11.0)	63 (10.2)
Former smokers	369 (61.2)	359 (58.8)	369 (60.7)	395 (63.7)
Non-smokers	165 (27.4)	183 (29.9)	172 (28.3)	162 (26.1)
Blood pressure (mm Hg)				
systolic	133.1 ± 21.4	134.1 ± 21.9	133.5 ± 22.2	132.6 ± 20.0
diastolic	83.6 ± 13.2	84.0 ± 12.9	84.0 ± 13.7	82.6 ± 12.1
Body mass index (Kg/m^2^)	27.6 ± 4.4	27.5 ± 4.0	27.7 ± 4.0	27.5 ± 3.8
Waist/Hip ratio	0.95 ± 0.07	0.95 ± 0.07	0.95 ± 0.07	0.95 ± 0.08

* m ± SD, except if specified

Groups are comparable for all the studied characteristics.

## Comments

The SU.FOL.OM3 Study was designed and initiated to test the hypothesis that supplementation with a natural form of folate (5-methyl-THF), a and/or n-3 fatty acids given in a population of subjects with past history of cardio or cerebro-vascular diseases might reduce fatal or non fatal ischemic events.

Human observational epidemiological studies and experimental works provide strong indication that n-3 PUFA, by one hand, and B-vitamins, on the other hand, could prevent cardiovascular diseases [29-32]. There are several randomized double blind interventions, testing n-3 fatty acids with available results or still on going. However, we should point out that some of these studies involve a small number of subjects; some of these studies has an open intervention design and used unblended clinical endpoint assessments. Some intervention trials with mostly intermediate end-points did not have a study design enabling a firm conclusion. Concerning trials with n-3 fatty acids, some studies used global dietary intervention and it is difficult to attribute the resulting effect only to n- 3 fatty acids. Even the large GISSI trial [21] was limited in that it did not use a placebo group but only a control group.

Secondary intervention trials with hard endpoints and B-vitamin supplementation have been launched in the last decade and first results do not provide clear evidence of any beneficial effect on CVD risk of lowering homocysteine concentration [14-17]. The hypothesis of different beneficial effects of folic acid on coronary and cerebrovascular prevention is still controversial. Indeed, a recent meta analysis has shown that a cerebrovascular protection effect was still associated with folic acid [33], while another-one did not demonstrate any reduction in cardiovascular risk [34]. Most of the previously published randomised controlled trials in the scope of homocysteine took place in regions where flour is fortified with folic acid. Such a fortification decreases plasma levels of homocysteine at the population level, and then, decreases the expected beneficial cardiovascular effect of folic acid supplementation. The decreased power of previous therapeutic trials could be an explanation for their negative results. The SUFOLOM 3 trial takes place in a non fortified zone, and the baseline relatively high levels of homocysteine (14 μmol/l) increases the power of the study.

Moreover most of trials with hard endpoints used a combination of B vitamins and most trials used pharmacological doses. This has the disadvantage that the results will be difficult to translate into dietary advises. In addition, recent research has indicated that supplementation with 5-methyl tetrahydrofolate (5-methyl-THF), the most abundant natural folate vitamer, is safe and lowers homocysteine levels [35-37]. This form of folate, in contrast to folic acid, does not lead to circulating unmetabolized folic acid [38]. Unmetabolized folic acid is hypothesized to mask the hematological manifestations of a vitamin B12 deficiency, thereby predisposing subjects to irreversible neurological damage. Stable 5-methyl-THF was not available when other intervention studies started and therefore they all use folic acid.

Finally, definitive proof that supplementation with n-3 fatty acids and/or B-vitamins leads to reduced cardiovascular disease morbidity and/or mortality remains insufficient. The currently available intervention trials have reported that the strength of the association of homocysteine with risk of cardiovascular disease is probably weaker than was believed at the time that many of the trials were initiated. For all these reasons, we proposed introducing, in our planned trial on the effects of folates and vitamin B, a specific arm testing the effect of n-3 fatty acids.

High-quality trials in different types of populations and specifically testing n-3 fatty acids and folates (with correct allocation concealment, hard CV endpoints including sudden cardiac death, blinding of participants and health providers, and adequate duration) are needed to be able to reach a conclusion.

## Conclusion

The originality of the SU.FOL.OM3 study is that this trial included supplementation with B-vitamins or n-3 fatty acids, and stroke and myocardial infarction as hard endpoints. A more important difference between the SU.FOL.OM3 study and the available published trials is the dose issue and the form of folates that is tested. All trials used pharmacological doses of B-vitamins and SU.FOL.OM3 is the only one trial that tests nutritional doses. It is also the only one to use 5-methyl THF instead of folic acid, which is predominantly present in circulation and the most abundant natural folate form. Several secondary prevention trials will be able in the next few years to answer the question whether a lower tHcy concentration through vitamin (folic acid, vitamin B6 and B12) and n-3 fatty acids suppementation have an effect on "hard" end points, like CHD and stroke.

## Competing interests

The authors declare that they have no competing interests.

## Authors' contributions

SB, JB and SC participated in the design of the study and performed the statistical analysis, PG and SH conceived of the study, and participated in its design and coordination and helped to draft the manuscript. All authors read and approved the final manuscript.

## Appendix

### THE SU.FOL.OM3 STUDY GROUP AND KEY PERSONNEL

- U557 Inserm/U1125 Inra/Cnam/University Paris 13, Centre de Recherche en Nutrition Humaine Ile-de-France

*Principal investigators: *Pilar GALAN, Jacques BLACHER, Sébastien CZERNICHOW, Serge HERCBERG

*Data Management*: Sandrine BERTRAIS, Fréderik CASSOR, Nathalie ARNAULT, Gwenael MONOT, Mohand AIT OUFELLA, Paul FLANZY,

- Ecole de Santé Publique, Faculté de Médecine, Université de Nancy

*Principal investigators: *Serge BRIANCON

-Laboratoire de Biochimie – Biologie Moléculaire – Nutrition – Métabolisme, CHU de Brabois, Vandoeuvre les Nancy

*Principal investigators: *Jean-Louis GUEANT, NAMOUR Bernard

- Laboratoire de Biochimie, CHR de Grenoble

*Principal investigators: *Véronique DUCROS, François LAPORTE, Alain FAVIER,

### SU.FOL.OM3 SCIENTIFIC COMMITTEE

Pierre Amarenco (Hôpital Lariboisière, Paris), Jacques Blacher (Hôpital Hôtel-Dieu, Paris), Eric Bruckert (CHU Pitié-Salpètrière, Paris), Nicolas Danchin (Hôpital Georges Pompidou, Paris), Pierre Ducimetière (Inserm, Villejuif), Christian Funk-Brentano (CHU St Antoine, Paris), Francois Gueyffier (CIC Hôpital Louis Pradel, Lyon), Jean-Louis Mas (Hôpital St Anne, Paris), François Paillard (CHU, Rennes), Christophe Tzourio (Inserm, Paris), Daniel Thomas ((CHU Pitié-Salpètrière, Paris)

### SU.FOL.OM3 FOLLOW-UP COMMITTEE

Pierre Amarenco (Hôpital Lariboisière, Paris), Valérie Assuerus (Association des Neurologues Libéraux de Langue Française), Jacques Beaune (Hôpital Louis Pradel, Lyon), Jacques Boulliat (Hôpital Bourg en Bresse), Mireille Brochier (CHU, Tours), Eric Bruckert (CHU Pitié-Salpètrière, Paris), Nicolas Danchin (Hôpital Georges Pompidou, Paris), Jean-François Dartigues (CHU Bordeaux), Jacque Delarue (CHU de Brest), Karine Demuth (Hôpital Georges Pompidou, Paris), Pierre Ducimetière (Inserm, Villejuif), Véronique Ducros (CHU, Grenoble), Alain Favier (CHU Grenoble), Jean Gardette (Pierre Fabre, Castres), Jean-Pierre Giordanella (CPAM Paris), Alain Grynberg (INRA, Paris), Jean-Louis Guéant (CHU, Nancy), Jean-Claude Guilland (CHU Dijon), Louis Guize (Hôpital Georges Pompidou, Paris), Marc Hommel (CHU, Grenoble), Olivier Joyeux (Centre hospitalier, Valence), Gérard Jullien (Collège National des Cardiologues, Marseille), Michel Krempf (CHU, Nantes), Denis Lairon (Inserm, Marseille), Gisèle Le Moël (Hôpital Bichat, Paris), Alain Lemoine (Centre hospitalier, Nevers), Didier Leys (CHU, Lille), Jean-René Lusson (CHU, Clermont-Ferrand), Jean-Louis Mas (Hôpital St Anne, Paris), Nicole Moatti (Hôpital Georges Pompidou, Paris), Jean-Pierre Monassier (Hôpital E. Muller, Mulhouse), Philippe Moulin (CHU, Lyon), François Paillard (CHU, Rennes), Isabelle Queré (CHU, Montpellier), Alberto Rhigetti (Hôpital Cantonal de Genève), Rosa-Maria Rodriguez (CHU, Nancy), Monique Romon-Rousseau (CHU Lille), Marie-Pierre Sauvant (CRNH, Clermont-Ferrand), Jean-Louis Schlienger (CHU, Strasbourg), Chantal Simon (CHU, Strasbourg), Daniel Thomas (CHU Pitié-Salpètrière, Paris), Christophe Tzourio (Inserm, Paris), François Viallet (Centre Hospitalier du Pays d'Aix, Aix en Provence)

### COMMITTEE FOR VALIDATION OF CARDIOVASCULAR EVENTS

Dr Jacques Blacher, Hôpital Hôtel-Dieu, Paris ; Serge Briançon, Ecole de Santé Publique, Nancy ; Yves Cottin, Hôpital du Bocage, Dijon ; Pierre Ducimetière, Unité INSERM 780, Villejuif ; Louis Guize, Hôpital Georges Pompidou, Paris ; Jacques Henry, Clinique de l'Orangerie, Strasbourg ; Jean-Jacques Monsuez, Hôpital René Muret-Bogottini, Sevran ; François Paillard, Hôpital Pontcaillou, Rennes

### COMMITTEE FOR VALIDATION OF NEUROVASCULAR EVENTS

Marc Bataillard, Hôpital André Boulloche, Montbeliard ; Gérard Besson, Hôpital Michallon, Grenoble ; Thomas De Broucker, Hôpital Delafontaine, St Denis; Christophe Tzourio, Hôpital Bichât, Paris; Thierry Rosolacci, Hôpital Sambre Avesnois, Maubeuge.
